# Geometric morphometric wing analysis represents a robust tool to identify female mosquitoes (Diptera: Culicidae) in Germany

**DOI:** 10.1038/s41598-020-72873-z

**Published:** 2020-10-19

**Authors:** F. G. Sauer, L. Jaworski, L. Erdbeer, A. Heitmann, J. Schmidt-Chanasit, E. Kiel, R. Lühken

**Affiliations:** 1 Aquatic Ecology and Nature Conservation, Carl von Ossietzky University Oldenburg, 26111 Oldenburg, Germany; 2 Bernhard Nocht Institute for Tropical Medicine, WHO Collaborating Centre for Arbovirus and Hemorrhagic Fever Reference and Research, 20359 Hamburg, Germany; 3 Faculty of Mathematics, Informatics and Natural Sciences, Universität Hamburg, 20148 Hamburg, Germany

**Keywords:** Entomology, Phylogenetics, Classification and taxonomy

## Abstract

Accurate species identification is the prerequisite to assess the relevance of mosquito specimens, but is often hindered by missing or damaged morphological features. The present study analyses the applicability of wing geometric morphometrics as a low-cost and practical alternative to identify native mosquitoes in Germany. Wing pictures were collected for 502 female mosquitoes of five genera and 19 species from 80 sampling sites. The reliable species identification based on interspecific wing geometry of 18 landmarks per specimen was tested. Leave-one-out cross validation revealed an overall accuracy of 99% for the genus and 90% for the species identification. Misidentifications were mainly due to three pairings of *Aedes* species: *Aedes annulipes* vs. *Aedes cantans*, *Aedes cinereus* vs. *Aedes rossicus* and *Aedes communis* vs. *Aedes punctor.* Cytochrome oxidase subunit I (COI) gene region was sequenced to validate the morphological and morphometric identification. Similar to the results of the morphometric analysis, the same problematic three *Aedes*-pairs clustered, but most other species could be well separated. Overall, our study underpins that morphometric wing analysis is a robust tool for reliable mosquito identification, which reach the accuracy of COI barcoding.

## Introduction

Mosquitoes (Diptera: Culicidae) are a widespread taxonomic group, which occurs from tropical to subarctic regions. Worldwide, 3578 mosquito species are currently known^1^. The females of most species are obligate hematophagous and thereby are important vectors and reservoirs of pathogens threatening billions of people all over the world. Global warming and globalization facilitate the spread and emergence of several agents of disease^2,3^. Outbreaks of West Nile virus (WNV)^4,5^ or chikungunya virus^6^ highlight the relevance of mosquito-borne diseases in Southern Europe. Furthermore, the ongoing Usutu virus circulation^7–10^ as well as the recent outbreaks of WNV^11^ underpin the increasing risk of mosquito-borne diseases also in Central Europe.

Vector capacity varies between different mosquito species, e.g. species-specific host preferences^12^ or vector competence^13^. Accordingly, accurate species identification is the prerequisite to understand patterns of pathogen transmission. Mosquitoes are commonly identified by morphology, e.g. Becker et al.^14^. Thereby, the identification requires considerable knowledge about the variation of the different taxonomic characters. In addition, important morphological characteristics such as legs or scales are often missing for field sampled specimens, hampering an accurate identification.

Geometric morphometrics is a promising alternative technique to identify insect species using anatomical landmarks^15^. Landmark collections are a cost-effective technique, which requires very little entomological experience compared to standard morphological identification. Its application increased distinctly in mosquito studies during the last two decades^16^. Hereby, usually the wings are used for the morphometrical diagnosis. Mosquitoes’ wing geometry is related to species-specific wing beat frequencies, which mediate the assortative mating behaviour^17^. In addition, the two-dimensional shape and the natural anatomical junctions of wing veins are ideal for the collection of landmarks^16^. It was shown before that the analyses of landmark configurations can be used to reliably identify mosquito species^18–20^, even if morphological identification with standard taxonomic keys is not possible e.g., female *Culex pipiens pipiens* form *pipiens* Linnaeus vs. *Culex torrentium* Martini^21^. However, the usage of morphometric wing analyses is largely underrepresented in European mosquito research. To the authors’ knowledge, only three studies applied morphometric wing analysis to native mosquito species in Europe. Intraspecific variations of mosquito wings were analysed for European population of *Aedes vexans* (Meigen)^22^ and *Cx. pipiens* s.l.^23^. Börstler et al.^21^ used morphometric diagnosis to discriminate female *Cx. pipiens* s.s. and *Cx. torrentium*.

The here presented study gives a comprehensive evaluation of this low-budget tool for the identification of a broad range of mosquito species. The morphometric wing characteristics for 19 of the most common mosquito species of Germany were analysed to evaluate the usability for species discrimination. In addition, we sequenced the COI gene to validate morphological and morphometric identification.

## Methods

### Mosquito sampling

**Figure 1:**
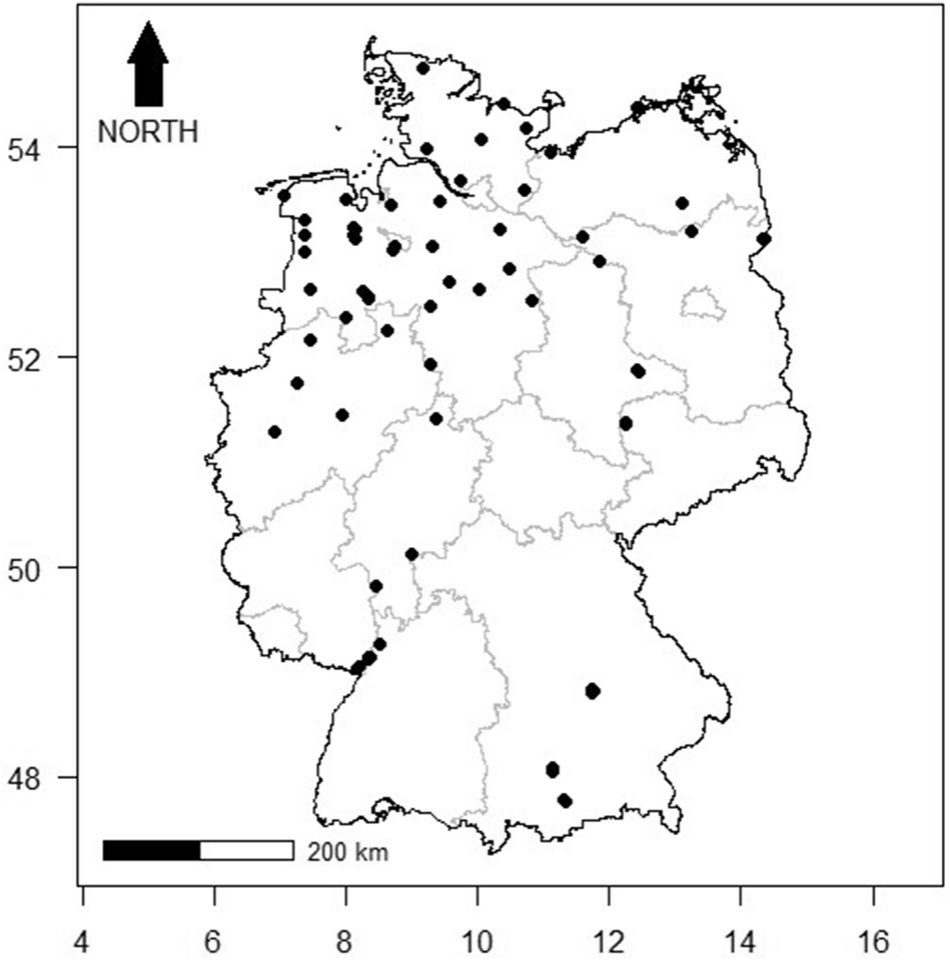
Mosquito sampling sites in Germany (black points). Latitude and longitude are based on the coordinate reference system WGS84.

The right wings of 502 females of 19 mosquito species were analysed (Table 1). Varying environmental conditions can influence the intraspecific shape and size of mosquito wings^24,25^. Hence, specimens were collected at 80 study sites (Fig. 1), covering different landscapes of Germany to capture a broad morphological variation per species. Mosquitoes were sampled over the seasons of 2017 and 2018. Adults were sampled with two different methods: CO_2_-baited Biogents-traps (Biogents, Regensburg, Germany) or hand-made aspirators modified from Vazquez-Prokopec et al.^26^. Immature stages were sampled with a standard dipper (Bioquip, CA, USA) in breeding sites and subsequently reared to adults in the laboratory. All specimens were identified by morphology^14,27^ and stored at − 18 °C in a freezer until further analysis. Specimens of each species were selected from at least three different sampling sites (Table 1). The wing pictures including coordinates of the sampling location, sampling date and sampling method for each specimen are given in the supplementary material (10.5061/dryad.zs7h44j5s).

**Table 1:** Mosquito species, abbreviations, number of sampling sites per species and number of specimens (N) used in this paper.

Scientific name	Abbreviation	Sampling sites	N
*Aedes annulipes* (Meigen, 1830)	Ae_ann	13	30
*Aedes cantans* (Meigen, 1818)	Ae_can	15	30
*Aedes caspius* (Pallas, 1771)	Ae_cas	6	17
*Aedes cinereus* Meigen, 1818	Ae_cin	11	30
*Aedes communis* (de Geer, 1776)	Ae_com	12	29
*Aedes geniculatus* (Olivier, 1791)	Ae_gen	13	24
*Aedes punctor* (Kirby, 1837)	Ae_pun	13	30
*Aedes rossicus* Dolbeskin, Gorickaja and Mitrofanova 1930	Ae_ross	3	14
*Aedes rusticus* (Rossi, 1790)	Ae_rust	12	29
*Aedes sticticus* (Meigen, 1838)	Ae_stic	14	30
*Aedes vexans* (Meigen, 1830)	Ae_vex	16	30
*Anopheles claviger* (Meigen, 1804)	An_clav	16	30
*Anopheles messeae* Falleroni, 1926	An_mess	13	19
*Anopheles plumbeus* (Stephens, 1828)	An_pb	13	30
*Coquillettidia richiardii* (Ficalbi, 1889)	Cq_rich	11	30
*Culex pipiens pipiens* form *pipiens* Linnaeus, 1758	Cx_pip	5	18
*Culex territans* (Walker, 1856)	Cx_terr	13	22
*Culiseta annulata* (Schrank, 1776)	Cs_ann	19	30
*Culiseta morsitans* (Theobald, 1901)	Cs_mors	17	30

### Measuring wing shape

The right wing of each specimen was removed and mounted under a cover slip (15 × 15 mm) with Euparal (Carl Roth, Karlsruhe, Germany). Pictures of each wing were taken under 20× magnification with a stereomicroscope (Leica M205 C, Leica Microsystems, Wetzlar, Germany). Fiji^28^ as bioscience package of ImageJ^29^ was used to digitize 18 landmarks (Figure S1). The landmark selection was in accordance with other studies analysing mosquito wing morphometry^18–20,30^. The wing pictures were divided among two observers (authors LE and FGS) and digitalised in random order to minimize a memory biased landmark collection between the mosquito specimens of the same species. One month later, the measurement was repeated for three specimens per species by four observers (authors LE, LJ, RL and FGS) to assess the degree of observer error and repeatability in landmark collection^31^.

### Genetic identification

DNA isolation was performed from the whole mosquito body, except of the right wing. Individual specimens were placed into 2 ml tubes and about 10 pieces of 2.0 mm zirconia beads (BioSpec Products, Bartlesville, USA) as well as 1 ml of cell culture medium (high-glucose Dulbecco’s modified Eagle’s medium; Sigma-Aldrich, St. Louis, MO, USA) were added. The homogenization was performed with a Tissuelyser II (Qiagen, Hilden, Germany) for 2 min at 30 oscillations/s and 200 μl of the homogenate were used for DNA extraction, which was performed with KingFisher Flex Magnetic Particle Processor using MagMAX CORE Nucleic Acid Purification Kit (both Thermo Fisher Scientific, Waltham, MA, USA). Polymerase chain reaction (PCR) amplification of cytochrome oxidase subunit I (COI) gene region was conducted with the protocol published by Fang et al.^32^ using the primers by Folmer et al.^33^. Sanger sequencing was applied for all positive amplicons (LGC Genomics, Berlin, Germany). Furthermore, morphologically identified *Cx. pipiens* s.l. and *An. maculipennis* s.l. specimens were typed to species level (*Cx. pipiens pipiens* form *pipiens* resp. *Anopheles messeae*) using two molecular assays^34,35^.

### Statistical analysis

Generalised Procrustes analysis of the two-dimensional landmark data set was performed to create aligned Procrustes coordinates and centroid size of each specimen with the R package geomorph^36^. The centroid size is defined as the square root of the sum of squared distances between centroid and each landmark^37^ and can be used as a proxy for wing size^38^. Mean difference in centroid size per species were compared by an analysis of variance (ANOVA), followed by pairwise comparisons of species using t-tests with Bonferroni-adjusted p-values. During the Procrustes analysis, raw landmark coordinates were centred, scaled and rotated, so that the resulting Procrustes coordinates describe the wing shape in itself^39^. Allometric effects of the centroid size on wing shape were assessed by the “procD.allometry” function from the geomorph package^36^ using 500 permutations. Wing shape variables were analysed by linear discriminant analyses (LDA) to classify genus and species, respectively (R package MASS^40^). Subsequently, each specimen was reclassified by a leave-one-out cross validation in order to test the classification obtained by the discriminant analysis. The mean shape configuration of the landmarks was used to visualize shape differences between the genera. In addition, a neighbour joining tree (NJ) was constructed to display the Mahalanobis distances between species means in a canonical variate analysis, using the functions “CVA” in Morpho^41^ and “nj”, “boot.phylo” and “drawSupportOnEdge” in ape^42^. Robustness of nodes was assessed by 1000 bootstrap replicates. To assess the degree of observer error, the shape variance due to the repeated measurements per specimen was compared with the mean shape variance per species of the original data set without repeated landmark collections. Therefore, shape variances were calculated by the “morphol.disparity” function in geomorph^36^ using 500 permutations. Furthermore, we assessed the fidelity of morphological characterization of our landmark data set through a Landmark Sampling Evaluation Curve (LaSEC) by using the function of Watanabe^43^. All analyses and visualisation including the R package ggplot2^44^ were carried out in the R environment^45^.

Sequences were pre-processed with Geneious 9.1.8 (Biomatters, Auckland, New Zealand). COI sequences were trimmed to 550 bp. To assess the phylogenetic relationship of the mosquito species identified in this study, a maximum likelihood tree was constructed using functions of the R packages seqinr^46^, ape^42^ and phangorn^47,48^. The HKY + G model was identified as the best-fit model of nucleotide substitution by the phangorn’s “modelTest” function based on calculations of Akaikeʼs information criterion. Robustness of nodes was assessed by 1000 bootstrap replicates. The COI sequences generated in this study were deposited in the GenBank database under the accession numbers MT731082–MT731276.

## Results

**Figure 2:**
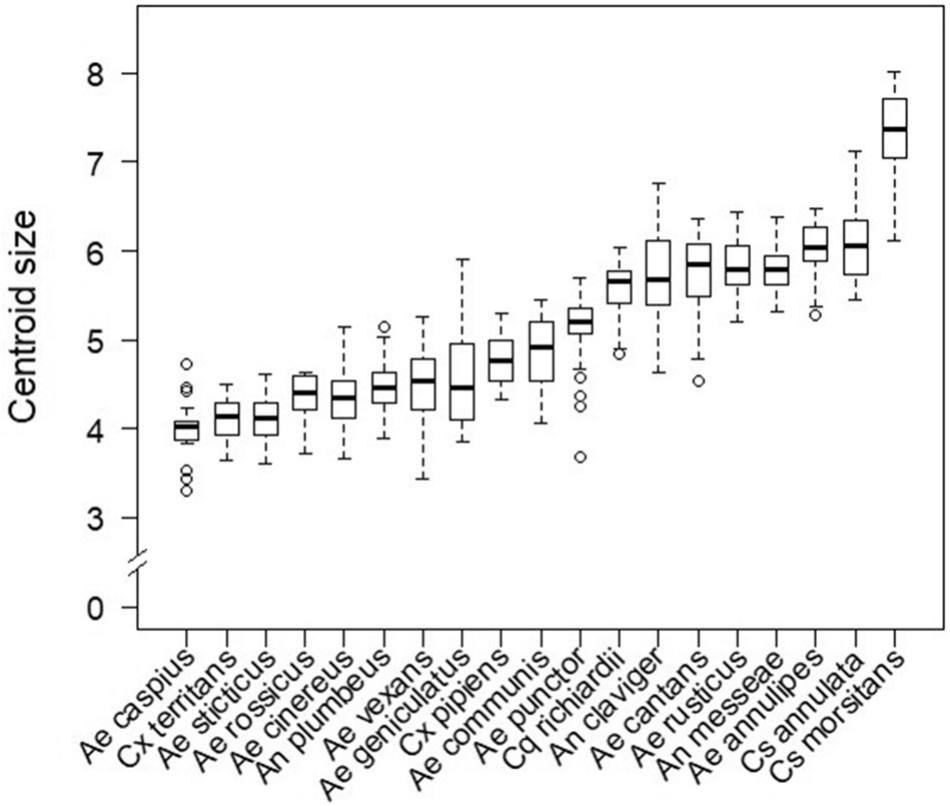
Boxplots showing variation of centroid size per species in ascending order. The centroid size is displayed as non-dimensional estimator for the wing size derived from the 18 landmark coordinates.

**Figure 3:**
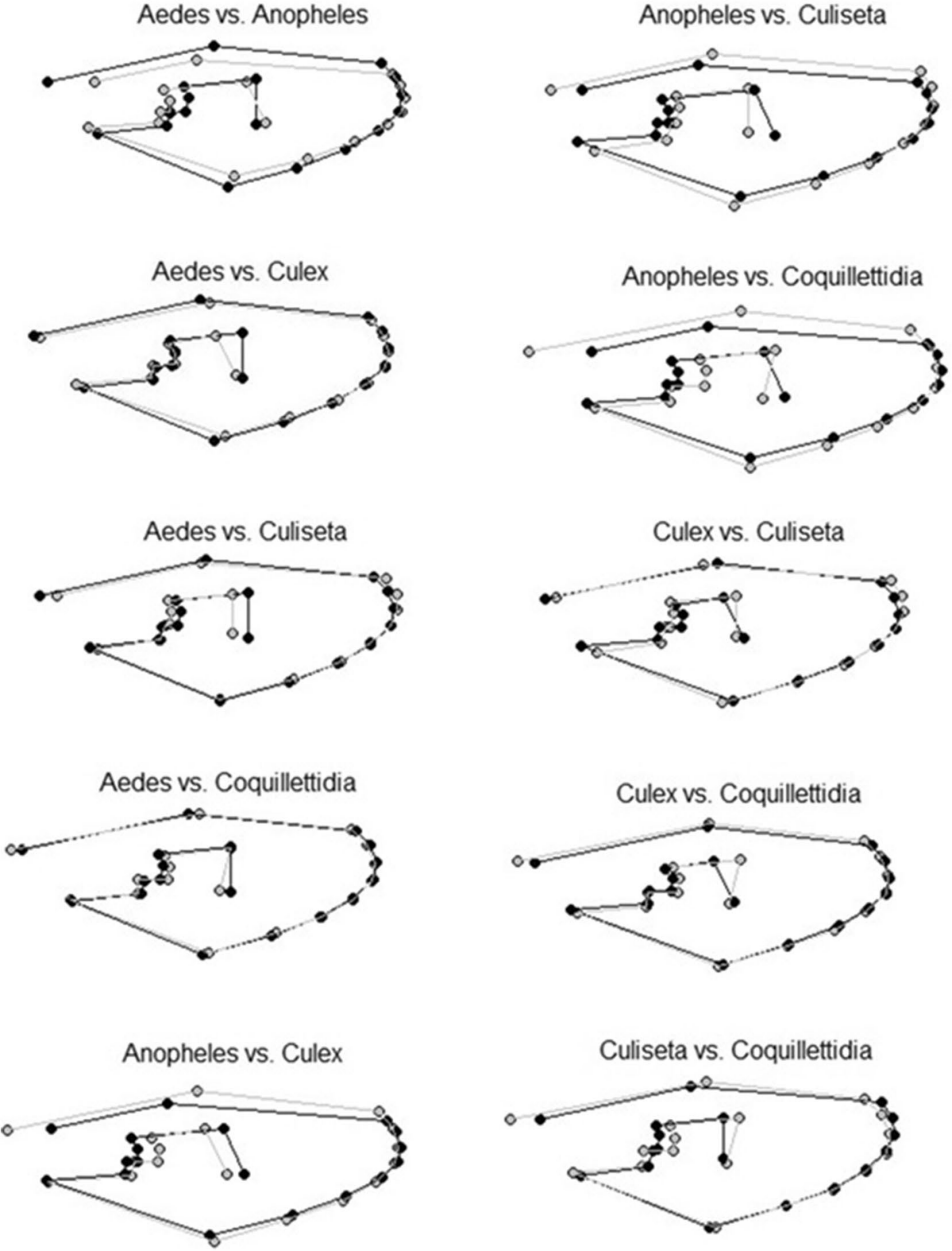
Comparison of the mean shape configurations between each genus pair. The first mentioned genus is shown in black and the second mentioned genus is shown in grey.

**Figure 4:**
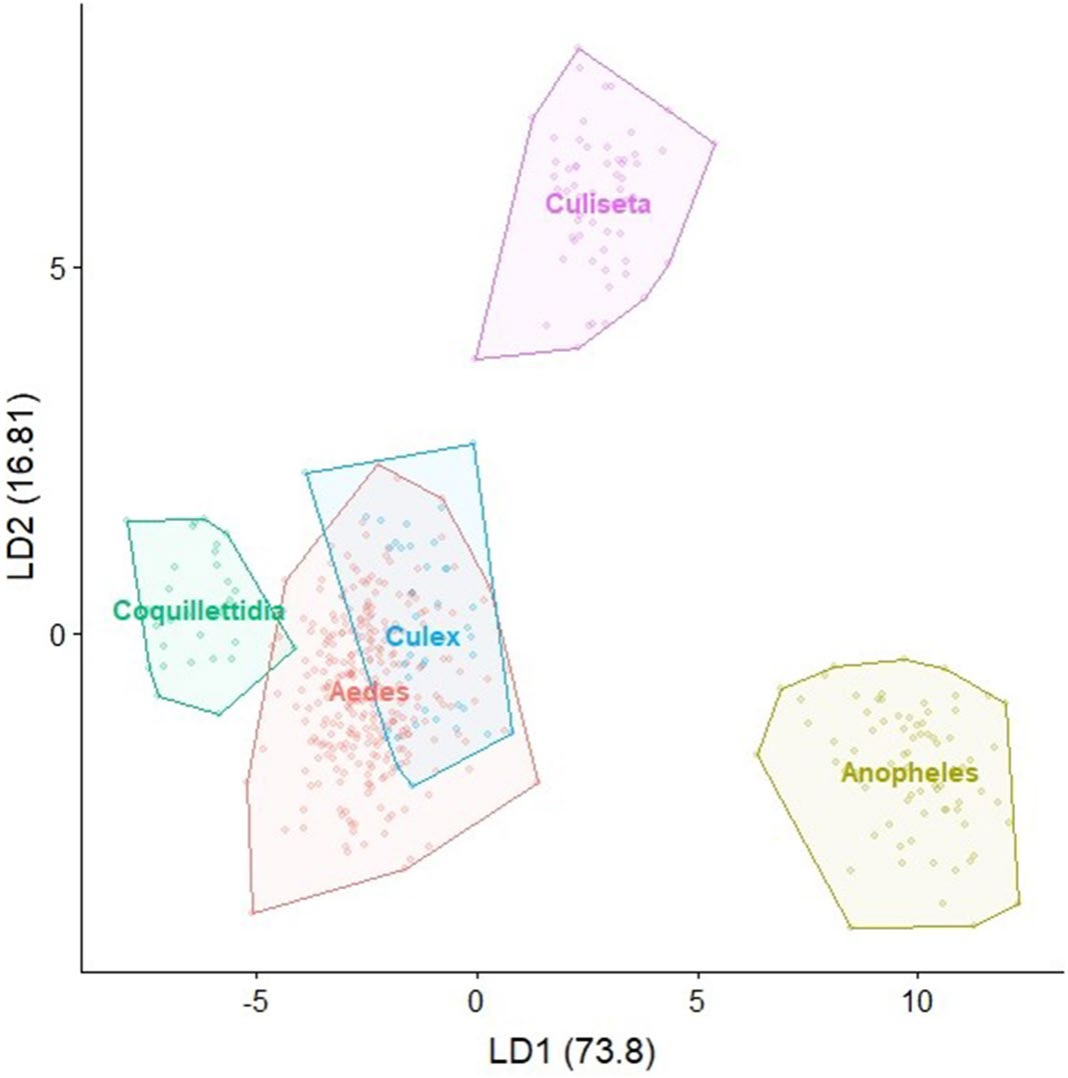
Visualization of the LDA showing the wing shape variation for the five analysed genera.

**Figure 5:**
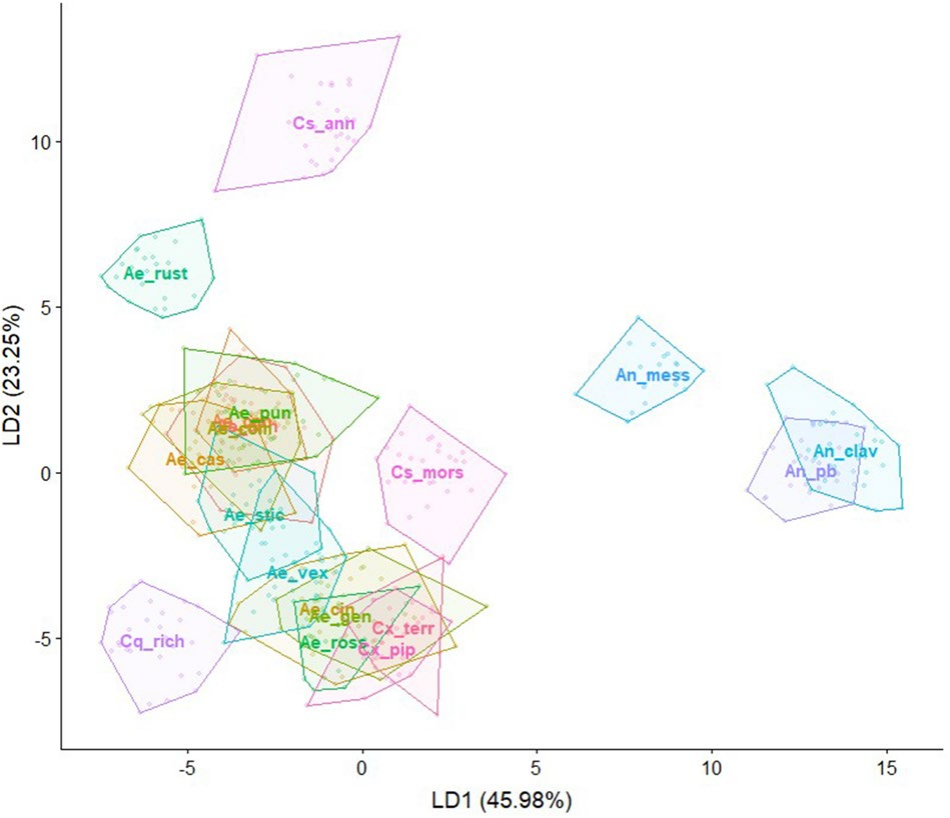
Visualization of the LDA showing the wing shape variation for the 19 species. Species abbreviations are given in Table 1. Species labels are displayed at the calculated mean centroid of the first two discriminants. The labels of *Ae. punctor, Ae. communis*, *Ae. cantans* and *Ae. annulipes* strongly overlap.

**Figure 6:**
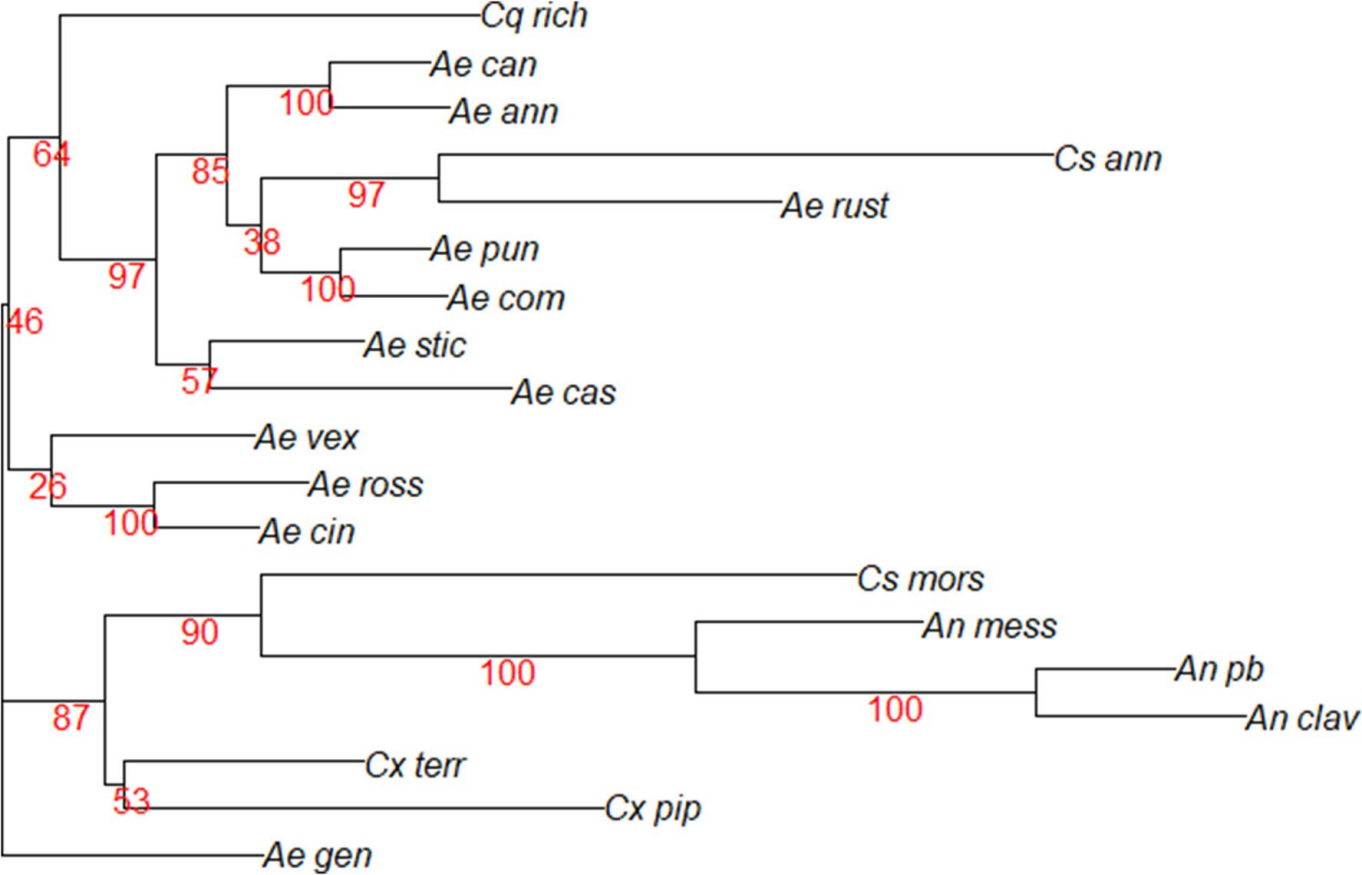
Neighbor joining tree for the species means derived from a canonical variate analysis. The tree was inferred from a Mahalanobis distance matrix (1000 bootstrap replicates). Branch support values are displayed in red numbers.

The mean centroid size differed significantly among the mosquito species (ANOVA, F = 143.3, p < 0.001). Highest centroid size was observed for *Culiseta morsitans* (Theobald) with almost twice the centroid size compared to the smallest species in our study: *Aedes caspius* (Pallas), *Aedes sticticus* (Meigen) and *Culex territans* (Walker) (Fig. 2). A small, but significant allometric effect was observed (R^2^ = 3.7%, p = 0.002). However, allometric residues were not removed since size variations were considered to be important for the species identification process^21^. A pairwise cross-validated reclassification test (leave-one-out method) to compare the five genera (*Aedes*, *Anopheles*, *Coquillettidia*, *Culiseta* and *Culex*) revealed an accuracy greater 99% (Fig. 4). Hereby, the genus of three out of 502 specimens was misidentified. One *Aedes* specimen was falsely identified as *Coquillettidia* and two *Culex* specimens were misidentified as *Aedes.* Differences in the shape variation were strongest for *Anopheles* in contrast to other genera (Figs. 3 and 4). In addition, the superimposed landmark coordinates of the junctions of radius 2 and 3 as well as media 1 and 2 varied most obviously when comparing the further genera (Fig. 3). Species identification accuracy based on the pairwise reclassification test was 90%. The error rate of 10% was mainly due to the comparisons of three pairings: *Aedes annulipes* (Meigen) vs. *Aedes cantans* (Meigen), *Aedes cinereus* Meigen vs. *Aedes rossicus* Dolbeskin, Gorickaja and Mitrofanova and *Aedes communis* (de Geer) vs. *Aedes punctor* (Kirby) (Table 2). In addition, the centroid size of these pairs did not differ significantly (Table S1). Other species were reclassified with a high reliability between 94 and 100% (Table 2, Fig. 5). The NJ tree resulted in high bootstrap values for most species, including the three pairings with a high error rate in the pairwise reclassification test (Fig. 6). While members of the genus *Culex* and *Anopheles* clustered together, species from the genus *Aedes* and *Culiseta* clustered heterogeneously over the NJ tree. The landmark collections of three randomly selected specimens per species were repeated by four different observers. The mean shape variance per specimens due to the different observers was 0.00047. This was considerably lower than the mean shape variance per species (0.00123) in the original landmark data set without repeated measurements, thus indicating a relatively small observer bias for the analysis of species-specific shape variations. The LaSEC did not suggest oversampling of landmarks in our data set (Figure S2).

**Table 2:**
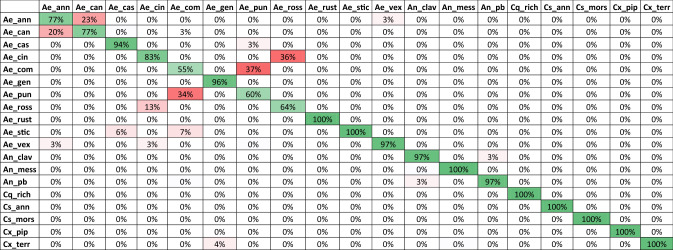
Species reclassification rates in percent calculated by a cross validation test (leave-one-out method).

Green cells highlight accurate reclassification. Red cells highlight incorrect reclassification. A list of species abbreviations is given in Table 1.

**Figure 7:**
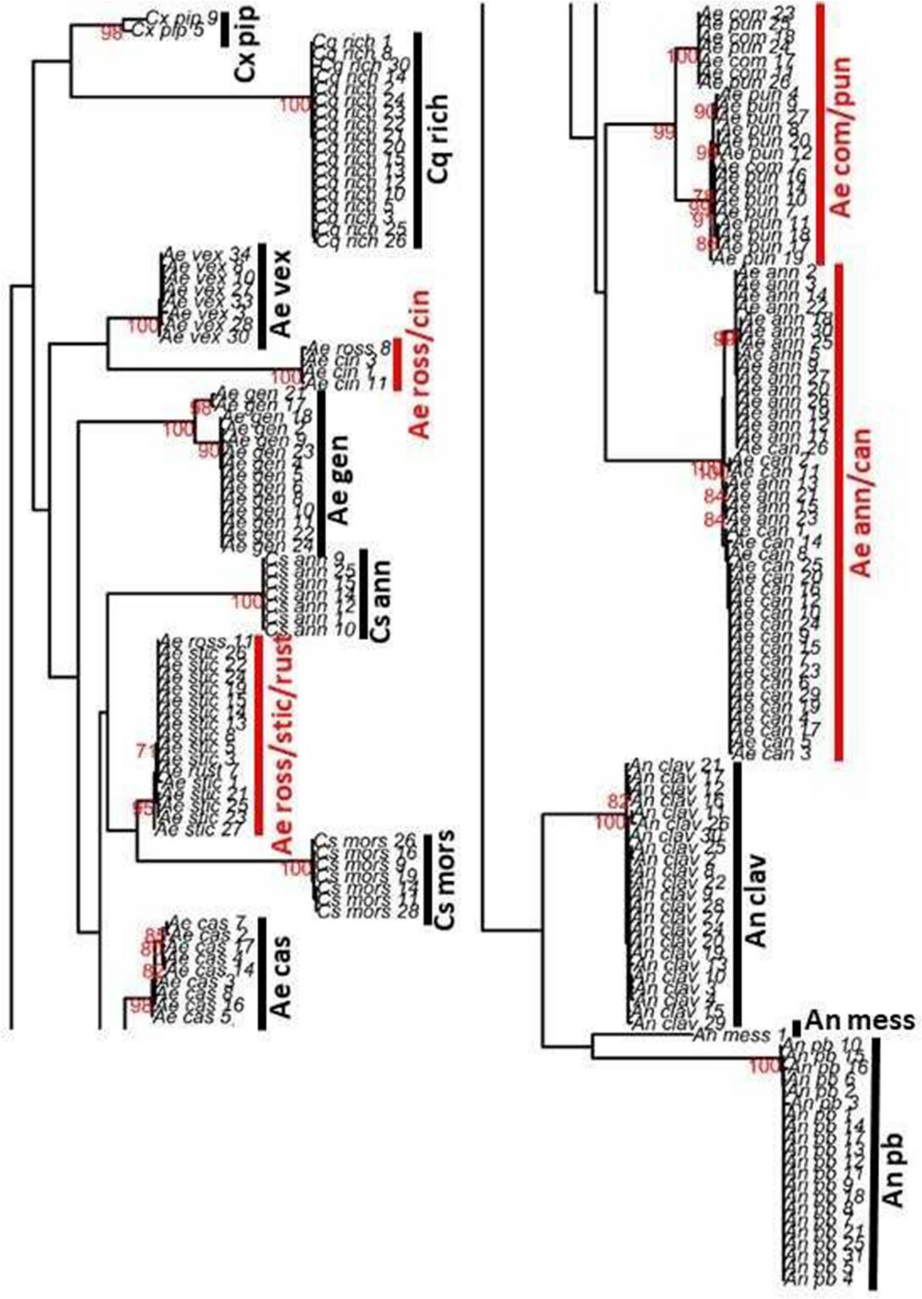
Maximum-likelihood phylogeny of the COI gene sequences. The tree was inferred using an HKY + G model (1000 bootstrap replicates). Branch support values of ≥ 70% are displayed in red numbers. Species groups with ambiguous clustering are highlighted in red. A list of species abbreviations is given in Table 1.

High quality, clean sequences were available for 195 mosquito specimens, covering 18 of the 19 taxa only missing the species *Cx. territans* (Fig. 7). In order to perform a verification of the consistency of the morphological and morphometric classification of the mosquito specimens, a maximum likelihood phylogenetic tree was constructed (Fig. 7). Most species clustered seperately. However, this was not the case for several members of the genus *Aedes*. The two species-pairs *Ae. annulipes* vs. *Ae. cantans* or *Ae. communis* vs. *Ae. punctor* did not cluster separately. In addition, the three sequences available for *Ae. rossicus* and *Aedes rusticus* (Rossi) either grouped with sequences of *Ae. cinereus* (*Ae. rossicus* only) or *Ae. sticticus* (*Ae. rossicus* and *Ae. rusticus*)*.*

## Discussion

We provided an overview of centroid size variation of the common mosquito species in Germany. As a multidirectional size, the centroid size is more sensitive to detect sample variation compared to one-dimensional measurements, e.g. wing length^49^. Moreover, it is an adequate indicator for the wing and body size^38^ and thus supply useful background information for the discrimination of certain species pairs as we could show in a pairwise comparison of the centroid size per mosquito species. However, accurate species identification solely based on wing size is not feasible^50^. The analysis of the superimposed shape coordinates produced by the Generalised Procrustes analysis is by far more informative for species identification^51^.

The results of the linear discriminant analysis based on the wing shape variables confirmed the high reliability of mosquito species identification through geometric wing morphometrics. The five most common mosquito genera in Europe (*Aedes*, *Anopheles*, *Coquillettidia*, *Culiseta* and *Culex*) are distinguishable by their wing shape. Our reclassification accuracy of 99% is in line with Wilke et al.^18^, who compared the wings of the genera *Aedes*, *Anopheles* and *Culex* by using the same combination of landmarks as in this study. A high reclassification accuracy was also observed on the species level, except three species pairs within the genus *Aedes*: *Ae. annulipes* vs. *Ae. cantans*, *Ae. cinereus* vs. *Ae. rossicus* and *Ae. communis* vs. *Ae. punctor*. Although the Mahalanobis distances of the species means derived from a canonical variate analysis clustered together in the NJ tree, an accurate differentiation of individual specimens among these three species pairs by their wings only does not seem appropriate. This is also reflected in the analysis of the COI sequences of these species. As demonstrated before, the species identification of mosquitoes using DNA barcoding of the COI gene can give ambiguous results. This in particular applies to members of the genus *Aedes* or *Culex*^52,53^. One explanation might be a close evolutionary relationship between the species, which is reflected in a high similarity of the wing geometry as shown for members of the genus *Anopheles*^19,54^. At least the females of these species pairs are morphological similar and difficult to distinguish by classical taxonomic features. In addition, these pairs are characterized by a similar breeding ecology and are often found in sympatry^14^. Further research is required for a clear distinction between these three pairs of *Aedes* species. This should include a multidisciplinary approach combining biological, ecological and molecular information^55^.

Shape and size of mosquito wings are not only species-specific. Geographically separated populations of the same species can have significant intraspecific variation in their wing morphology^22,56,57^. Furthermore, abiotic and biotic conditions of breeding habitats can influence the wing geometry through carry-over effects from the immature to the adult stage^58–61^. Those factors have to be kept in mind when choosing mosquito specimens for the analyses of species-specific wing patterns. Specimens from a single sampling location would probably reduce shape variation per species, thus leading to an overestimation of the actual interspecific variation. Therefore, for each species we used specimens from at least three different sampling locations to capture a broad wing shape variance. Nevertheless, our results revealed a high reliability to distinguish mosquito species and so underpins the robustness of morphometric wing diagnosis as tool for mosquito identification, which is in line with Henry et al.^62^, who successfully discriminated specimens of *Aedes aegypti* (Linnaeus) and *Aedes albopictus* (Skuse) sampled from different parts of the world.

Besides natural differences between wing from mosquito specimens of the same species, morphometric analysis can be influenced by different measurement errors^63^. Inter-observer variation of landmark collection is likely the most important source of errors. Hence, we compared the measurement by four observers revealing a small, but considerable observer bias. In particular, when interested in finer intraspecific shape variations this might influence the results potentially leading to misinterpretations. One reason for the observer variation is probably the presence of wing scales on the veins, which can impede the precise landmark collection^50^. The removal of the wing scales before taking pictures may reduce observer variation and should be considered in future studies, but also increase the effort of data collection. However, completely observer unbiased measurement results are impossible. Therefore, as suggested by Dujardin et al.^64^, we provide the wing pictures in a way third parties can use them for own analyses including metadata on the sampling location and date of sampling (10.5061/dryad.zs7h44j5s), e.g. allowing the investigation of intraspecific wing patterns in future studies.

Our study supports the applicability of geometric wing morphometrics as a complementary technique for mosquito identification, which has certain advantages over other methods. Geometric morphometric is less expensive and time-consuming compared to genetic sequencing^21,65^, e.g. representing a low-cost routine alternative under semi-field conditions. In comparison with classical taxonomical identification it requires less experience, thus representing a useful tool to control own morphological identification, e.g. for inexperienced observers. Furthermore, specimens can be identified even if only one wing is preserved or if mosquitoes are stored or sampled in fluids (e.g. ethanol), which commonly result in a loss of scales relevant for accurate morphological identification of most female mosquitoes.

In conclusion, most mosquito species chosen for this study could be distinguished reliably by geometric wing morphometrics. The accuracy obtained by geometric wing morphometrics was comparable to the results of COI barcoding, but the morphometric analysis of wings is less expensive and time-consuming to implement. Hence, wing morphometrics has a high potential to complement species identification in future entomological studies or mosquito surveillance programs in Europe.

## Supplementary information

Supplementary Information 1.

## Data Availability

Data for the wing pictures are stored in a repository and are available at 10.5061/dryad.zs7h44j5s.
