# Supplementary material for: Geometric morphometric wing analysis represents a robust tool to identify female mosquitoes (Diptera: Culicidae) in Germany
Source: Sci Rep. 2020 Oct 19;10:17613. doi: 10.1038/s41598-020-72873-z (PMC7573584; doi:10.1038/s41598-020-72873-z)
Supplement: Supplementary file 1 — Supplementary Information 1. [file 41598_2020_72873_MOESM1_ESM.docx]

**Supplementary material**

**Title**

Geometric morphometric wing analysis represents a robust tool to identify female mosquitoes (Diptera: Culicidae) in Germany

**Authors**

Sauer F.G.^1^, Jaworski L.^1,2^, Erdbeer L.^1^, Heitmann A.^2^, Schmidt-Chanasit, J.^2,3^, Kiel E^1^, Lühken R^2,3^

^1^Carl von Ossietzky University Oldenburg, Aquatic Ecology and Nature Conservation, 26111 Oldenburg, Germany,

^2^Bernhard Nocht Institute for Tropical Medicine, WHO Collaborating Centre for Arbovirus and Hemorrhagic Fever Reference and Research, 20359 Hamburg, Germany

^3^Universität Hamburg, Faculty of Mathematics, Informatics and Natural Sciences, 20148 Hamburg, Germany

**Corresponding authors**

Felix G. Sauer, [felix.sauer@uol.de](mailto:felix.sauer@uol.de)

Renke Lühken, [renkeluhken@gmail.com](mailto:renkeluhken@gmail.com)

Figure S1: Wireframe with the 18 landmarks and example pictures of the wings with marked landmarks.

Figure. S2: Landmark Sampling Evaluation Curves (LaSEC)

Table S1: P-values of a pairwise comparison of the centroid size of mosquito species

Datadryad: Original pictures of each wing with metadata (https://doi.org/10.5061/dryad.zs7h44j5s)


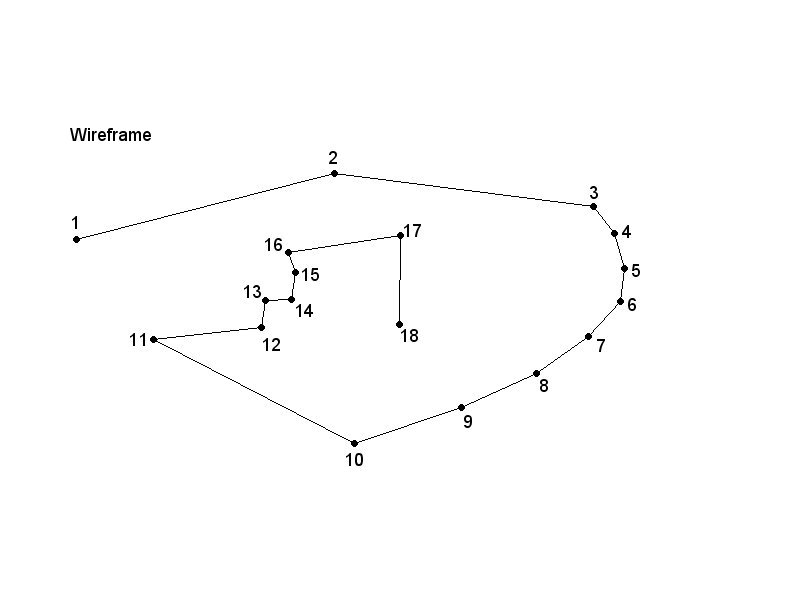


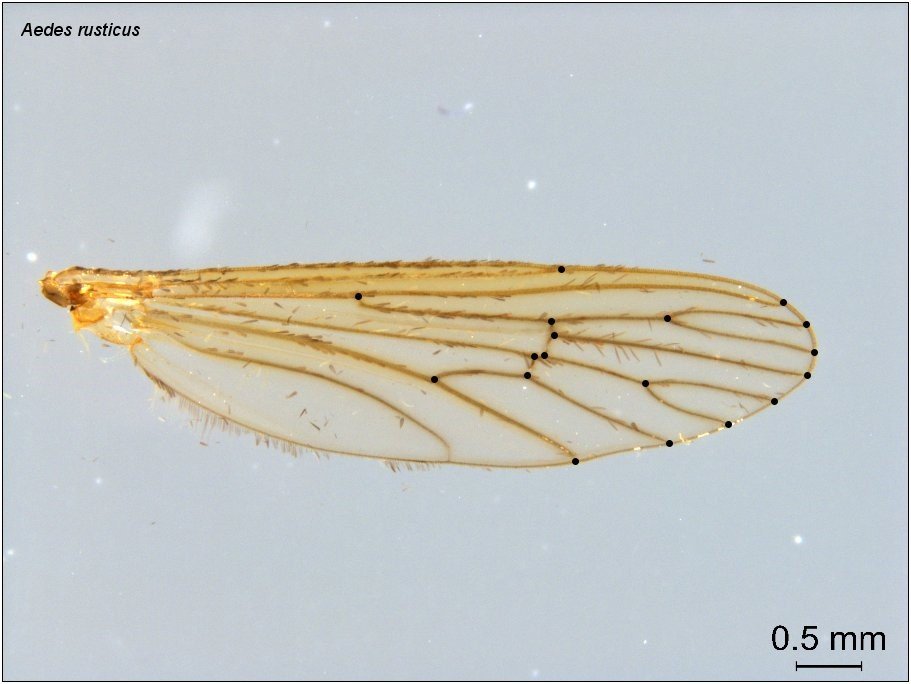


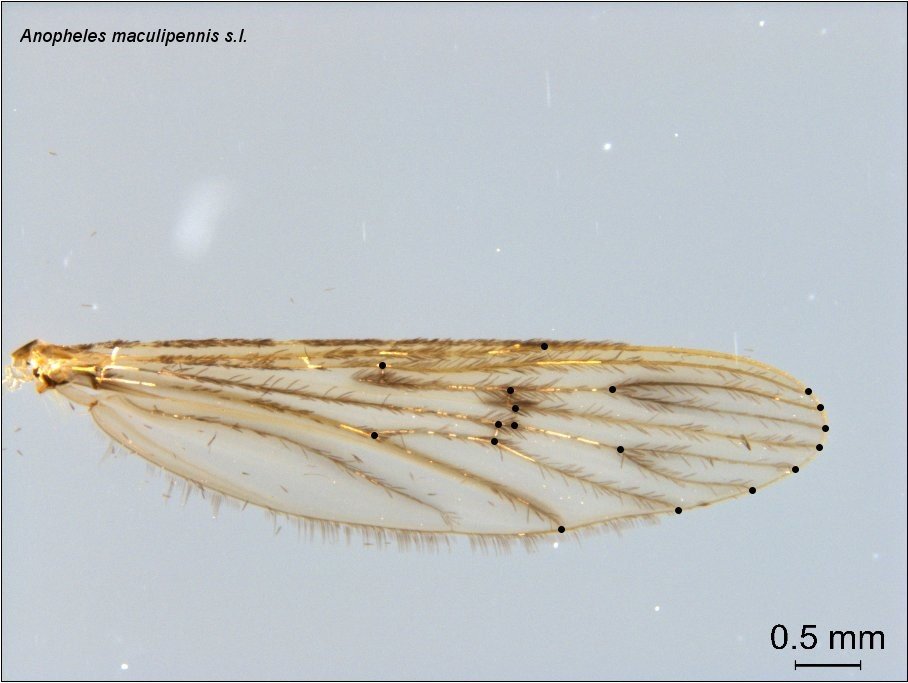

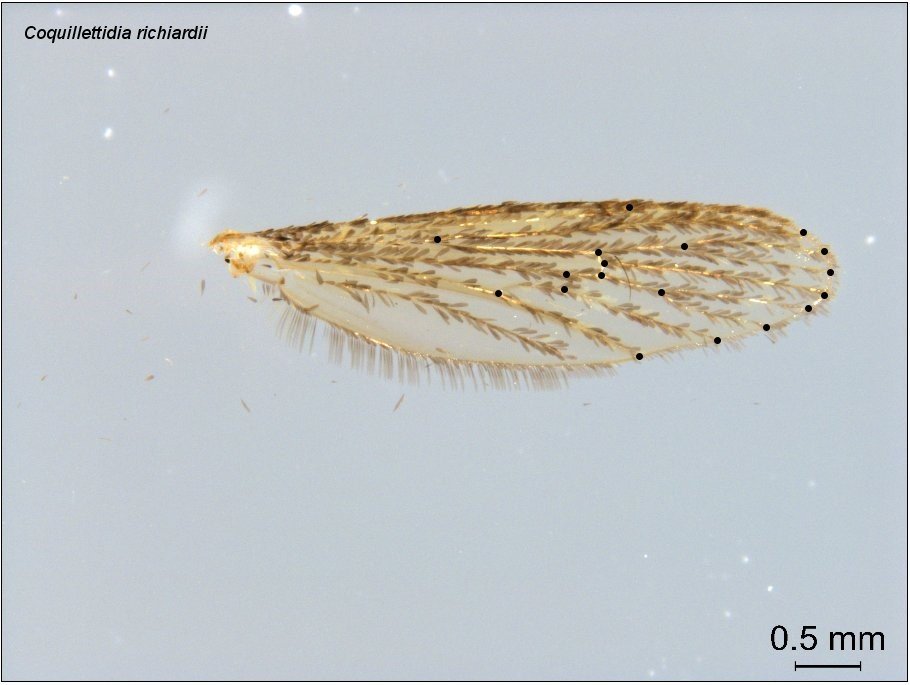

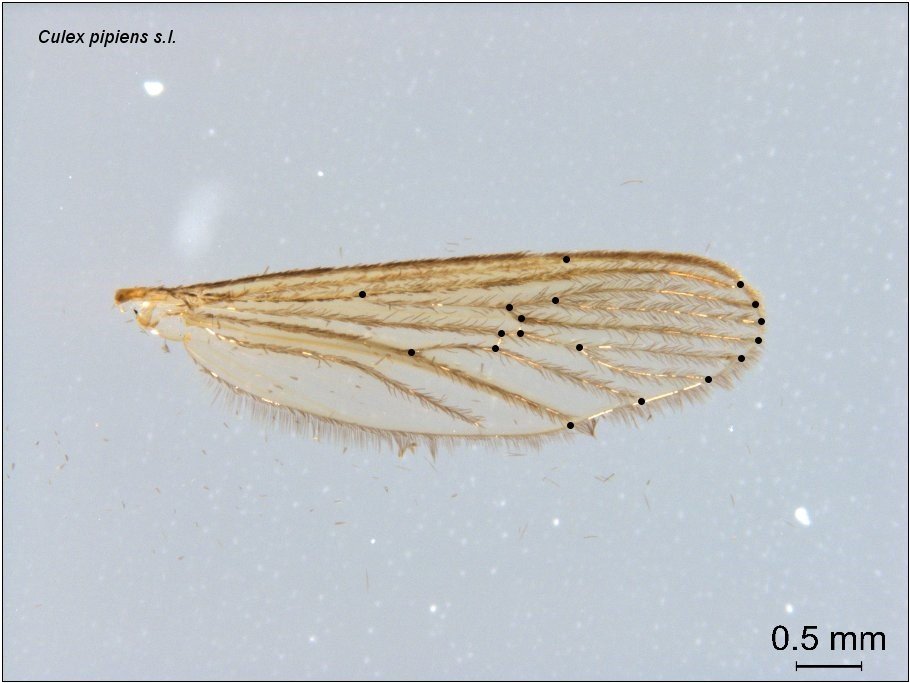

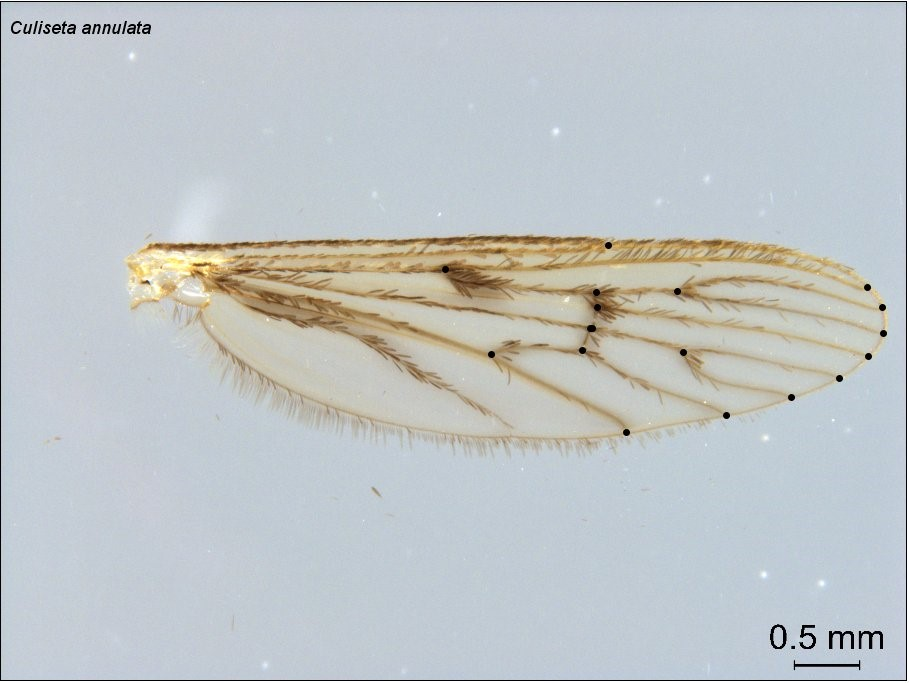


**Figure S1:** The wireframe of the landmark selection and exemplary wing pictures with landmarks.

**Figure S2.** Landmark sampling evaluation curve (LaSEC) to assess the fidelity of morphological characterization by the 18 wing landmarks of the 526 specimens used in this study. Grey lines indicate values from one iteration of subsampling (= 1000). Dark line indicate median fit value: fit = 0.9: 13 landmarks, fit = 0.95: 15 landmarks, fit = 0.99: 17 landmarks.


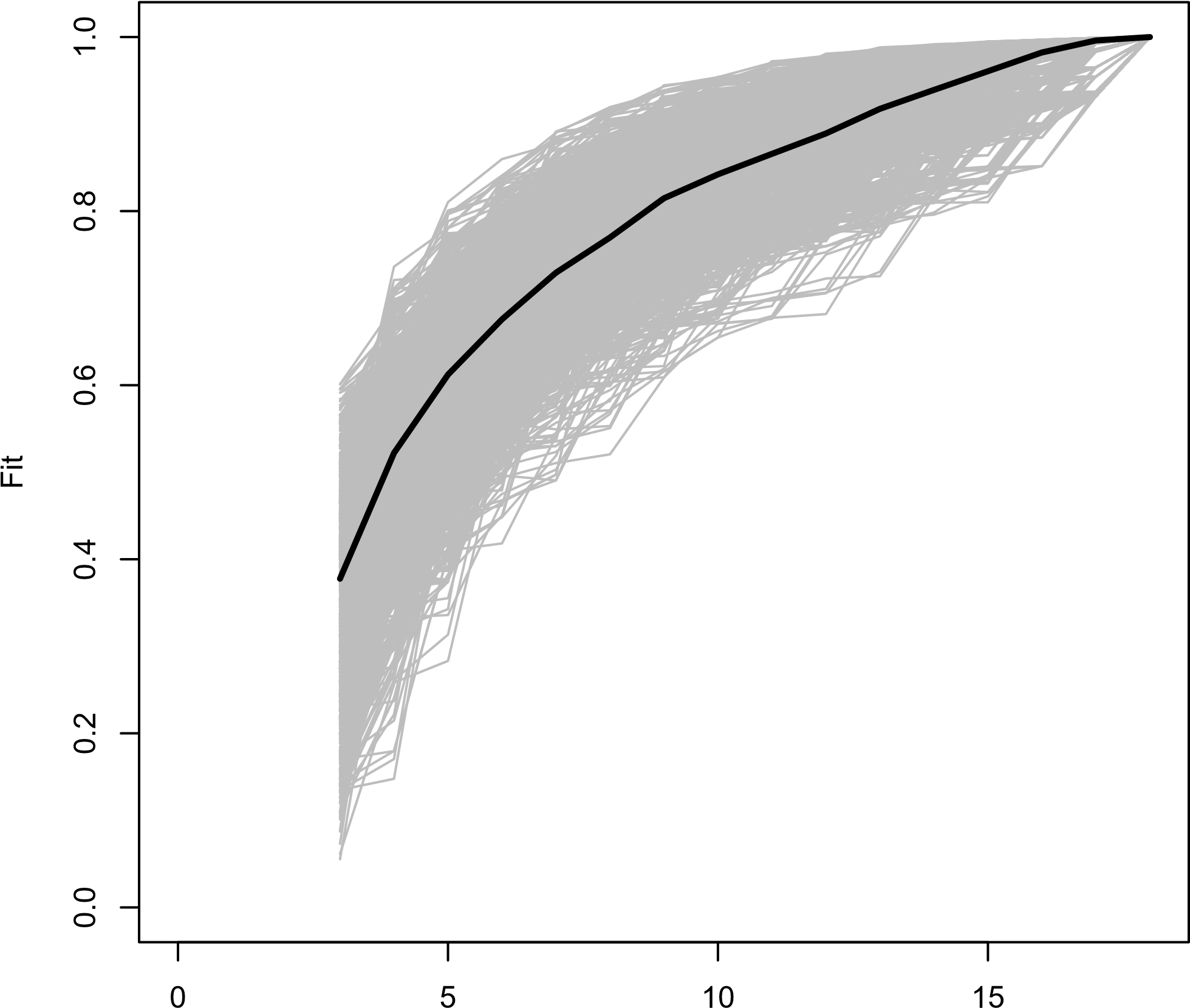


Number of landmarks


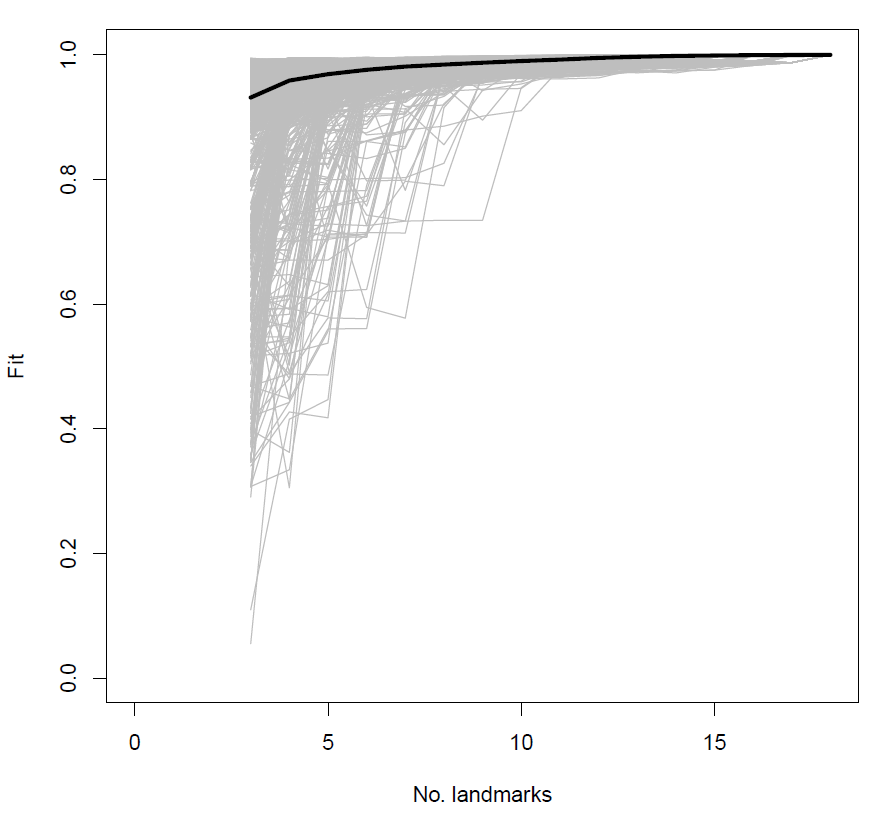


Number of landmarks

**Figure S3.** Landmark sampling evaluation curve to assess the number of landmarks needed to characterize centroid size variation reliably. Grey lines indicate values from one iteration of subsampling (= 1000). Dark line indicate median fit value.

**Tab S1:** P-values of pairwise comparison of mosquito wings’ centroid size per species calculated by t-tests and Bonferroni-adjusted. A list of abbreviations is given in Tab. 1.

|  | **Ae_ann** | **Ae_can** | **Ae_cas** | **Ae_cin** | **Ae_com** | **Ae_gen** | **Ae_pun** | **Ae_ross** | **Ae_rust** | **Ae_stic** | **Ae_vex** | **An_clav** | **An_mess** | **An_pb** | **Cq_rich** | **Cs_ann** | **Cs_mors** | **Cx_pip** |
| --- | --- | --- | --- | --- | --- | --- | --- | --- | --- | --- | --- | --- | --- | --- | --- | --- | --- | --- |
| **Ae_can** | 0.24 | - | - | - | - | - | - | - | - | - | - | - | - | - | - | - | - | - |
| **Ae_cas** | < 0.01 | < 0.01 | - | - | - | - | - | - | - | - | - | - | - | - | - | - | - | - |
| **Ae_cin** | < 0.01 | < 0.01 | 0.23 | - | - | - | - | - | - | - | - | - | - | - | - | - | - | - |
| **Ae_com** | < 0.01 | < 0.01 | < 0.01 | < 0.01 | - | - | - | - | - | - | - | - | - | - | - | - | - | - |
| **Ae_gen** | < 0.01 | < 0.01 | < 0.01 | 1 | 0.43 | - | - | - | - | - | - | - | - | - | - | - | - | - |
| **Ae_pun** | < 0.01 | < 0.01 | < 0.01 | < 0.01 | 1 | < 0.01 | - | - | - | - | - | - | - | - | - | - | - | - |
| **Ae_ross** | < 0.01 | < 0.01 | 1 | 1 | 0.01 | 1 | < 0.01 | - | - | - | - | - | - | - | - | - | - | - |
| **Ae_rust** | 1 | 1 | < 0.01 | < 0.01 | < 0.01 | < 0.01 | < 0.01 | < 0.01 | - | - | - | - | - | - | - | - | - | - |
| **Ae_stic** | < 0.01 | < 0.01 | 1 | 1 | < 0.01 | 0.01 | < 0.01 | 1 | < 0.01 | - | - | - | - | - | - | - | - | - |
| **Ae_vex** | < 0.01 | < 0.01 | < 0.01 | 1 | 0.05 | 1 | < 0.01 | 1 | < 0.01 | 0.02 | - | - | - | - | - | - | - | - |
| **An_clav** | 0.22 | 1 | < 0.01 | < 0.01 | < 0.01 | < 0.01 | < 0.01 | < 0.01 | 1 | < 0.01 | < 0.01 | - | - | - | - | - | - | - |
| **An_mess** | 1 | 1 | < 0.01 | < 0.01 | < 0.01 | < 0.01 | < 0.01 | < 0.01 | 1 | < 0.01 | < 0.01 | 1 | - | - | - | - | - | - |
| **An_pb** | < 0.01 | < 0.01 | 0.012 | 1 | 0.01 | 1 | < 0.01 | 1 | < 0.01 | 0.10 | 1 | < 0.01 | < 0.01 | - | - | - | - | - |
| **Cq_rich** | < 0.01 | 1 | < 0.01 | < 0.01 | < 0.01 | < 0.01 | < 0.01 | < 0.01 | 1 | < 0.01 | < 0.01 | 1 | 0.47 | < 0.01 | - | - | - | - |
| **Cs_ann** | 1 | 0.14 | < 0.01 | < 0.01 | < 0.01 | < 0.01 | < 0.01 | < 0.01 | 1 | < 0.01 | < 0.01 | 0.13 | 1 | < 0.01 | < 0.01 | - | - | - |
| **Cs_mors** | < 0.01 | < 0.01 | < 0.01 | < 0.01 | < 0.01 | < 0.01 | < 0.01 | < 0.01 | < 0.01 | < 0.01 | < 0.01 | < 0.01 | < 0.01 | < 0.01 | < 0.01 | < 0.01 | - | - |
| **Cx_pip** | < 0.01 | < 0.01 | < 0.01 | < 0.01 | 1 | 1 | 1 | 0.04 | < 0.01 | < 0.01 | 0.26 | < 0.01 | < 0.01 | 0.07 | < 0.01 | < 0.01 | < 0.01 | - |
| **Cx_terr** | < 0.01 | < 0.01 | 1 | 1 | < 0.01 | 0.02 | < 0.01 | 1 | < 0.01 | 1 | 0.05 | < 0.01 | < 0.01 | 0.17 | < 0.01 | < 0.01 | < 0.01 | < 0.01 |
